# Evaluation of implementation of patient involvement in Taiwan’s pharmaceutical reimbursement decision-making process

**DOI:** 10.1017/S026646232510038X

**Published:** 2025-07-29

**Authors:** Yi-Ling Tsai, Wen-Wen Yang, Grace Hui-Min Wu, Shih-Chang Lin, Chao-Hsiun Tang, Raoh-Fang Pwu

**Affiliations:** 1 Department of Health Care Management, National Taipei University of Nursing and Health Sciences, Taipei City, Taiwan; 2 Data Science Center, Fu Jen Catholic University, New Taipei City, Taiwan; 3 Department of Physical Therapy and Assistive Technology, National Yang Ming Chiao Tung University, Taipei City, Taiwan; 4 School of Health Care Administration, Taipei Medical University, Taipei City, Taiwan; 5 Health Outcomes and Technology Teaching and Education Alliance, Taipei City, Taiwan

**Keywords:** Patient involvement policy, Pharmaceutical reimbursement, Taiwan national health insurance, Mosaic model, Policy implementation and evaluation

## Abstract

**Objectives:**

Patient involvement enhances transparency, legitimacy, and responsiveness in pharmaceutical reimbursement decisions. Guided by the mosaic model, this study recognizes effective patient engagement requires diverse context-specific approaches. Despite Taiwan’s National Health Insurance Administration (NHIA) implementing policies, gaps remain between intent and practice. This study evaluates NHIA’s incorporation of patient inputs into reimbursement decisions and examines factors influencing involvement.

**Methods:**

We analyzed pharmaceutical company-initiated reimbursement submissions for catastrophic illnesses reviewed by the Pharmaceutical Benefit and Reimbursement Scheme Joint Committee (PBRS) from 2016 to 2023. Data sources included PBRS meeting records, the Online Patient Opinion Platform (OPOP), and NHIA notification E-mails. Generalized linear models identified predictors of patient involvement. The association between patient involvement and PBRS decisions was also explored.

**Results:**

Patient involvement occurred in 28.4 percent (80/282) of all submissions, increasing from 17 percent (2016) to 44 percent (2023). Despite aligning with OPOP criteria, patient involvement remained incomplete. Discussion-type submissions, oncology drugs, and new drug applications showed higher involvement, whereas autoimmune diseases and new indication submissions had lower involvement. Budget impact and innovation categories were not significant predictors in adjusted models. The presence of patient involvement was not significantly associated with the PBRS approval rate. Ad hoc analysis revealed increased involvement for new indications following policy expansion.

**Conclusions:**

Despite NHIA’s efforts, patient involvement implementation remains suboptimal. Structured mechanisms and expanded patient involvement beyond high-profile submissions and PBRS are crucial to broaden patient involvement. This study provides practical insights for East Asian healthcare systems advancing patient involvement amid limited empirical research.

## Background

Patient and public involvement (PPI) has gained increasing attention in healthcare decision-making, particularly health technology assessment (HTA) and pharmaceutical reimbursement decisions (1). PPI is regarded as essential for enhancing transparency, legitimacy, and responsiveness in policymaking, ensuring that healthcare decisions reflect patient needs and societal values rather than relying solely on technical and economic considerations (2–6). Many countries have integrated PPI into HTA processes, shifting the focus from necessity to strategies for optimizing practical implementation (7–10). Despite these advancements, achieving meaningful and systematic integration of patient input remains a persistent challenge.

### Evolution of patient and public involvement frameworks

Public participation in policymaking has been widely studied across disciplines, with Arnstein’s Ladder of Citizen Participation (11) being particularly influential. Arnstein categorized public participation into eight hierarchical levels, ranging from nonparticipation (manipulation and therapy) to tokenism (informing, consultation, placation), and ultimately citizen power (partnership, delegated power, citizen control) (11). Although Arnstein’s ladder provides a foundational framework for understanding power dynamics in participation, its linear hierarchy has limitations in the complex healthcare context, particularly in HTA processes where multiple stakeholders interact across various decision points.

Tritter and McCallum (2006) address these limitations by proposing a more flexible, nuanced ‘mosaic’ model (12). Unlike the ladder’s focus on power transfer, the mosaic model highlights the diverse nature of patient experiences and expertise, multiple motivations for involvement beyond power acquisition, the legitimacy of lay knowledge alongside technical expertise, the importance of both process and outcome in participation, and the dynamic, nonlinear nature of stakeholder relationships. The mosaic approach is particularly relevant to HTA processes, where patient involvement occurs at multiple points and through various mechanisms rather than following a single upward trajectory toward increasing patient power in involvement and engagement.

### Contemporary HTA patient and public involvement frameworks

Building on these theoretical foundations, contemporary HTA agencies commonly classify PPI into three categories that better reflect practical implementation of stakeholder involvement in healthcare decision-making (3;13–20):

1. Information (passive participation) – stakeholders receive updates on policy decisions without formal input. This aligns with the lower rungs of Arnstein’s ladder but, within the mosaic model, represents one legitimate form of engagement depending on context.
2. Consultation (interactive participation) – stakeholders provide feedback, but policymakers retain decision-making authority. This middle-ground approach allows for the integration of diverse patient perspectives (a key mosaic principle) while maintaining governance structures.
3. Collaboration (empowered participation) – stakeholders and policymakers jointly develop policies and share decision-making authority. This approach most closely realizes the mosaic model’s emphasis on diverse expertise and mutual learning.

Leading HTA systems, such as those in Canada, the UK, and Australia, have implemented collaborative patient involvement through early engagement initiatives, patient advisory panels, and codecision-making frameworks (19–23). These systems recognize that meaningful patient involvement requires multiple, interconnected mechanisms rather than a single approach – aligning with the mosaic concept of varied, context-specific participation.

### Patient and public involvement in Taiwan’s National Health Insurance (NHI)

Taiwan’s National Health Insurance Administration (NHIA) has primarily implemented patient and public involvement across all three engagement levels, though with an emphasis on Information and Consultation rather than full Collaboration. Viewing Taiwan’s approach through the mosaic model lens reveals a system with diverse engagement mechanisms that nonetheless lacks comprehensive integration of patient perspectives throughout the decision-making process.

1. **Information**: NHIA publicly announces coverage decisions on its website, press releases and social media platforms such as Facebook and Line@. Since June 2019, two patient representatives have been permitted to attend the PBRS (Pharmaceutical Benefit and Reimbursement Scheme Joint Committee) meetings as observers. These two representatives serve as general patient advocates, presenting the perspectives of relevant patient groups. However, they lack voting rights and may only contribute to discussions when explicitly invited by the committee chair (24). This approach, while providing transparency, represents a limited tile within the potential mosaic of engagement.
2. **Consultation**: In August 2016, the NHIA introduced the “NHI Operation Directions for Facilitating Patient Involvement in Pharmaceutical Reimbursement Decisions” (25) and launched the Online Patient Opinion Platform (OPOP) to collect patient comments on new drug submissions for catastrophic illnesses (25–27). In October 2021, the scope of OPOP was expanded to include applications for new indications, signalling a growing institutional commitment to patient and public involvement (28). These consultation mechanisms add additional tiles to the mosaic but remain segregated from core decision-making processes.
3. **Collaboration:** Among the 30 members of PBRS Committee, three represent insurance beneficiaries and three are employer representatives (29). The committee operates by consensus decision-making, thereby granting insurance beneficiary representatives and employer representatives equal decision-making authority regarding reimbursement coverage alongside other medical experts and healthcare providers. This represents the most advanced form of engagement in Taiwan’s system, though these representatives may not fully capture the specific experiences of patients with the conditions under consideration.

Although public representatives share authority in coverage decision-making in Taiwan, patient involvement remains relatively limited. Public interests primarily focus on the appropriate allocation of limited resources and maintaining a well-functioning health service. Patients, however, typically advocate for broader coverage and may hold positions that diverge from public representatives regarding increased public funding (30). This tension illustrates a key mosaic principle – that different stakeholders bring varied perspectives and priorities to the engagement process.

### The gap between patient involvement policy intent and implementation

Previous research has highlighted several gaps in the implementation of patient involvement (31;32). Patient representatives and advocacy groups have expressed concerns that not all eligible submissions for catastrophic illnesses are included in the Online Patient Opinion Platform (OPOP) (31). Moreover, even when patients provided opinions via OPOP, evidence indicates that their inputs were not consistently incorporated into the materials presented at PBRS meetings (31). Additionally, although patient representatives can attend PBRS meetings, their participation is restricted to an observation role (25;32).

### Study objectives

In light of concerns regarding the gap between policy intent and actual implementation, this study aimed to systematically evaluate the extent of patient involvement in Taiwan’s pharmaceutical reimbursement decision-making processes. Specifically, it focuses on the consultation stage, addressing two questions: (i) To what extent does the NHIA collect and integrate patient input into Taiwan’s reimbursement decision-making? (ii) What factors contribute to variations in patient involvement across different types of reimbursement submissions?

## Methods

### Study design

This study employed a retrospective observational design to analyze the implementation of patient involvement in Taiwan’s NHI pharmaceutical reimbursement decision-making processes from 2016 to 2023. This study’s evaluation approach was informed by Tritter and McCallum’s mosaic model (12), which conceptualizes patient involvement as a complex, multi-dimensional process rather than a linear progression. By examining multiple mechanisms of patient involvement (OPOP listings, email notifications, and PBRS meeting materials) across different submission types, this study captures the varied ‘tiles’ of Taiwan’s engagement mosaic rather than focusing solely on hierarchical power dynamics. Additionally, this study investigated the association between patient involvement and final PBRS decisions.

### Definition of patient involvement

Patient in this study includes:

1. Patient, “an individual with a disease or disorder who is using some aspect(s) of the healthcare system because of this disease or disorder” (30).
2. Consumer/user, “an individual who uses, has used, or intends to use a particular health technology or service” (30).
3. Carer/caregiver, “an individual who is the unpaid informal primary or secondary caregiver for a patient” (30).
4. Patient advocate, “an individual who represents and advocates for the interests of a particular group of patients on a committee” (30).

**In this study, patient involvement was defined as actions initiated by the NHIA rather than the voluntary sharing of opinions by the patients.** Patient involvement was identified when the NHIA (i) manually listed a submission on OPOP or sent email notifications to patient advocacy groups to solicit input or (ii) incorporated patient opinions into PBRS meeting materials, regardless of whether these inputs influenced final decisions.

Submissions in which patient representatives merely attended PBRS meetings without providing documented contributions were categorized as ‘Information’ rather than ‘Consultation’ and were excluded from the patient involvement analysis. This distinction highlights that patient involvement, as evaluated in this study, relies on proactive measures taken by the NHIA rather than voluntary actions by patients.

### Data collection and exclusion criteria

Data were collected from PBRS meeting minutes and materials, the OPOP website (26), and NHIA email notifications sent to patient advocacy groups. Submissions were included if they involved catastrophic illnesses listed in the PBRS records between 2016 and 2023, as these submissions were required to undergo the OPOP process based on the NHIA’s criteria for collecting patient input through OPOP. Catastrophic illnesses, as defined by the NHIA, comprise 30 categories, including cancers, hereditary coagulation disorders, systemic autoimmune syndromes, chronic mental illnesses, congenital metabolic disorders, and others (33).

Submissions were excluded if they (i) did not have a definitive PBRS decision by 2023 (e.g., submissions still under review) or (ii) fell outside the scope of OPOP, including price increase applications, modifications to clinical evidence, changes in administrative requirements, clarifications of reimbursement criteria, prescriber setting restrictions, revisions to registration requirements, biosimilars or generics, reimbursement terminations, managed entry agreement adjustments, or new specifications for existing indications.

### Selection of submission characteristics

The NHIA provides multidisciplinary meeting materials for the PBRS members, containing detailed submission information. These materials included the drug profile (e.g., manufacturer, dosage, disease area, and application type), comparative effectiveness, treatment landscape, budget impact analysis, reference drug prices from ten advanced countries, deliberation type, innovation category, patient opinions, HTA recommendations from Canada, Australia, and the UK, and proposed coverage decisions from expert consultation meetings (ECM).

From these materials, six key characteristics were prioritized for analysis based on expert consultation and their direct relevance to the NHIA’s decision-making process:

1. Year of PBRS conclusion: stratified into three periods to reflect the policy evolution. (i) 2016–2018: initial launch of OPOP; (ii) 2019–2021: a policy shift permitting patient representatives to observe PBRS meetings; (iii) 2022–2023: strengthening government commitment to patient involvement.
2. Deliberation type: submissions were categorized by the NHIA as follows: (i) discussion-type: requires extensive discussion; (ii) reporting type: expedited approval without extensive discussion unless objections arise (see Supplementary Appendix 1 for NHIA’s formal criteria (34)).
3. The disease area: categorized as (i) oncology: solid tumors and hematological cancers; (ii) rare diseases and hemophilia: combined in generalized linear models (GLMs) owing to their earmarked funding structure (further details in Supplementary Appendix 2); (iii) autoimmune diseases; (iv) other catastrophic illnesses.
4. Application type: defined as (i) new drug submissions: covering new compositions, dosage forms, administration methods, or therapeutic compounds. (ii) new indications: expanding the approved use of existing reimbursed drugs.
5. Drug innovation category: applicable only to new drug submissions, classified as (35): (i) category 1: breakthrough drugs; (ii) category 2A: improved drug; (iii) category 2B: similar drugs; (iv) not available (NA): new indications or drugs without an innovation classification.
6. Anticipated budget impact: defined based on estimates by the HTA team of the Center for Drug Evaluation for the 5th reimbursement year, viewed from the NHIA’s perspective (not manufacturer projections): (i) ≤ NTD 100 million (around USD 30,255): including cost-saving, budget-neutral, and unavailable estimates; (ii) > NTD 100 million (around USD 30,255): high financial impact.

These six factors were selected because they align with the NHIA’s core deliberation criteria, influence policy-level decision-making, and are hypothesized to impact the NHIA’s prioritization of patient involvement efforts. The rationale for selecting these submissions is summarized in Table 1.

**Table 1.** Submission characteristics and rationales of study inclusion

Characteristics	Definition/categories	Rationale for study inclusion
Year of PBRS conclusion	- **2016–2018**: Initial launch of OPOP; - **2019–2021**: Patient representatives observed PBRS meetings; - **2022–2023**: Strengthened government commitment to patient involvement.	Policy context may influence NHIA’s responsiveness to patient involvement.
Deliberation type	Categorized by NHIA: - **Discussion-type**: Requiring extensive discussion; - **Reporting-type**: Expedited approval without extensive discussion unless objections arise.	Discussion-type submissions may allow greater patient involvement.
Disease area	These catastrophic illnesses all fall in the scope of OPOP. - **Oncology** - **Rare diseases and hemophilia** - **Autoimmune diseases** - **Other catastrophic illnesses**	Disease areas may differ in advocacy strength or policy prioritization.
Application type	- **New drug**: New compositions, dosage forms, administration methods, and therapeutic compounds; - **New indication**: Expanding the approved uses of existing reimbursed drugs.	Patient involvement may vary between novel treatments and expanded indications.
Innovation category	Only applicable to new drug submissions, categorized by ECM. - **Category 1**: Breakthrough drugs; - **Category 2A**: Improved drugs; - **Category 2B**: Similar drugs; - **Not available (NA)**: For new indications or those new drugs without an innovation category.	Higher innovation levels may correlate with greater patient involvement.
Anticipated budget impact	**Anticipated budget impact in the 5th reimbursement year, evaluated by CDE/HTA** **- ≤ NTD 100 million (around USD 30,255)** **- > NTD 100 million (around USD 30,255)**	Submissions with higher financial implications may receive more NHIA attention.

Abbreviations: PBRS, Pharmaceutical Benefit and Reimbursement Scheme; OPOP: Online Patient Opinion Platform; NHIA, National Health Insurance Administration; ECM, expert consultation meetings; CDE/HTA, Center for Drug Evaluation/Health Technology Assessment Division.

### Outcome variables

1. Presence or absence of patient involvement: defined according to the abovementioned criteria.
2. Final PBRS reimbursement decisions: categorized as “approved” or “not approved”. The final PBRS decision was selected over the ultimate reimbursement realization to avoid potential biases arising from post-approval factors such as pricing negotiations or managed entry agreements.

### Ad-hoc analysis: impact of NHIA’s OPOP expansion

Since October 2022, the NHIA has expanded the scope of OPOP to include new indications, removing a previous barrier that limited patient involvement (28). To evaluate the immediate impact of this policy change, we conducted an ad hoc analysis comparing patient involvement rates for new indications before and after policy implementation. Chi-square tests evaluated differences in patient involvement rates whereas logistic regression assessed policy changes as predictors of patient involvement. This ad-hoc analysis aims to determine whether the procedural changes of NHIA led to measurable improvements in patient and public involvement. Owing to the limited postpolicy timeframe, the findings should be considered preliminary rather than conclusive.

### Data validation

A three-step validation process was conducted to ensure data accuracy: (i) structure check: variable definitions and categorizations were reviewed by four field experts; (ii) practice rule check: contentious cases were resolved through expert consensus meetings, conducted among authors and guided by the PBRS practice rules (Supplementary Appendix 3); and (iii) data entry check: all data set entries were cross-validated by at least two independent experts to eliminate errors.

### Statistical analysis

Descriptive statistics were used to summarize submission characteristics and patient involvement trends. Differences between submissions with and without patient involvement were examined using the chi-square test for categorical variables. Fisher’s exact test was used to ensure accurate statistical inferences when the sample sizes were small.

To quantify the association between submission characteristics and patient involvement, generalized linear models (GLMs) were employed to estimate relative risks (RRs) with 95 percent confidence intervals (CIs). GLMs were chosen over traditional logistic regression models because the odds ratios (ORs) from logistic regression can overestimate the likelihood of common outcomes when event rates exceed 10 percent. This consideration was particularly relevant in this study, where patient involvement was observed in 28.4 percent of the submissions. Using GLMs with a Poisson distribution and robust error variance, the analysis provided direct estimates of relative risk, allowing for more interpretable comparisons across submission types, disease areas, and application types.

The analysis was conducted in two stages: (i) a simple GLM to evaluate unadjusted associations between each predictor and patient involvement and (ii) a multiple GLM to adjust for potential confounders and assess independent effects while controlling for other variables. The models incorporated six key predictors outlined in the Selection of Submission Characteristics section. Statistical significance was set at *p* ≤ .05.

### Ethical considerations

This study was approved by the Institutional Review Board of National Yang Ming Chiao Tung University and deemed exempt from full review under the regulations for human subject research (IRB No.: NYCU112076AW).

## Results

### Characteristics of investigated submissions

**Figure 1.**
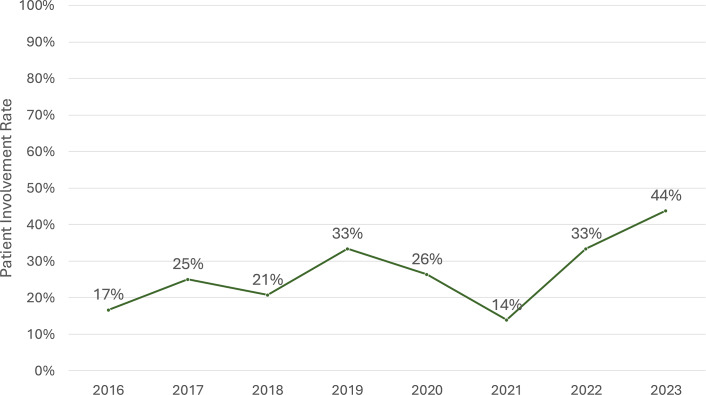
Trends in patient involvement implementation in Taiwan’s pharmaceutical reimbursement process.

A total of 282 reimbursement submissions for catastrophic illnesses met the inclusion criteria. Among these, 80 submissions (28.4 percent) documented patient involvement, as determined by listing on OPOP, NHIA E-mail notifications, or inclusion in PBRS meeting materials. Over the study period, patient involvement increased significantly from 17 percent in 2016 to 44 percent in 2023 (Figure 1). Although patient involvement rates temporarily declined to 14 percent in 2021, they rebounded to 33 percent in 2022 and reached 44 percent in 2023.

The characteristics of the investigated submissions are summarized in Table 2 and Supplementary Appendix Figure 1. Most submissions (64.2 percent) were of the discussion type, requiring extensive discussion in PBRS meetings. Oncology-related submissions accounted for the largest proportion (55.7 percent), followed by rare diseases (21.6 percent), autoimmune diseases (11.0 percent), and other catastrophic illnesses (5 percent). New drug submissions (52.8 percent) slightly outnumbered new indication applications (47.2 percent).

**Table 2.** Characteristics of the investigated submissions

Variable	Total submissions (% of total)	Patient involvement implemented *n* (% of the category)	*p*-value
**All submissions**	**282 (100%)**	**80 (28.4%)**	
**The year of the submission conclusion in PBRS**			
2016	30 (10.6%)	5 (16.7%)	*p* = .079
2017	24 (8.5%)	6 (25.0%)	
2018	29 (10.3%)	6 (20.7%)	
2019	54 (19.1%)	18 (33.3%)	
2020	38 (13.5%)	10 (26.3%)	
2021	29 (10.3%)	4 (13.8%)	
2022	30 (10.6%)	10 (33.3%)	
2023	48 (17.0%)	21 (43.8%)	
**Deliberation type**			
Discussion	181 (64.2%)	64 (35.4%)	***p* < .001**
Reporting	101 (35.8%)	16 (15.8%)	
**Disease area**			
Oncology	157 (55.7%)	47 (29.9%)	***p* = .040**
-Hematology	44 (15.6%)	15 (34.1%)	
-Solid tumor	113 (40.1%)	32 (28.3%)	
Hemophilia	19 (6.7%)	4 (21.1%)	
Rare disease	61 (21.6%)	24 (39.3%)	
Auto-immune disease	31 (11.0%)	3 (9.7%)	
Others	14 (5.0%)	2 (14.3%)	
**Application type**			
New drug	149 (52.8%)	80 (53.7%)	***p* < .001**
-New composition	131 (46.5%)	69 (52.7%)	
-New dosage form	7 (2.5%)	0 (0%)	
-New administration	7 (2.5%)	1 (14.3%)	
-New compound	4 (1.4%)	1 (25.0%)	
New indication	133 (47.2%)	9 (6.8%)	
**Innovation category (New drug only, *n* = 149)**			
Category 1 (breakthrough drugs)	25 (16.8%)	16 (64.0%)	***p* = .012**
Category 2A (improved drug)	59 (39.6%)	32 (54.2%)	
Category 2B (similar drug)	57 (38.3%)	18 (31.6%)	
Not available	8 (5.4%)	5 (62.5%)	
**The anticipated budget impact in the fifth reimbursement year**			
Cost-saving or no budget impact	86 (30.5%)	14 (16.3%)	***p* < .001**
≦ NTD 1 million (around USD 30,255)	91 (32.3%)	28 (30.8%)	
Between NTD 1 and 2 million (around USD 30,255 and 60,511)	32 (11.3%)	16 (50.0%)	
> NTD 2 million (around USD 60,511)	46 (16.3%) 27 (9.6%)	19 (41.3%) 3 (11.1%)	
Not available	27 (9.6%)	3 (11.1%)	

Among the 149 new drug submissions, 16.8 percent were classified as Category 1 (breakthrough drugs), 39.6 percent as Category 2A (improved drugs), and 38.3 percent as Category 2B (similar drugs). Regarding the anticipated budget impact, 27.6 percent of the submissions exceeded NTD 100 million (around USD 30,255), whereas the majority (62.8 percent) had lower financial implications.

### Predictors of patient involvement

**Figure 2.**
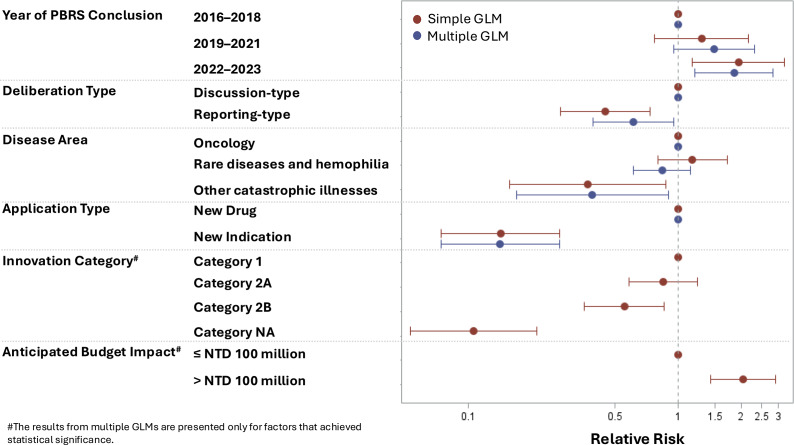
Forest plot of relative risk estimates from simple and multiple GLMs. GLM: generalized linear model.

The chi-square analysis results are summarized in Table 2, and the findings from the GLMs are presented in Figure 2. These analyses identified key submission characteristics associated with patient involvement. The data underlying Figure 2 are presented in Supplementary Appendix Table 4.

#### Submission year and type

Statistical analyses confirmed an increasing trend in patient involvement over time. The simple GLM indicated that submissions from 2019 to 2021 (RR: 1.29, 95 percent CI: 0.77–2.17) and 2022–2023 (RR: 1.94, 95 percent CI: 1.17–3.22) were more likely to involve patients compared to the 2016–2018 baseline. This trend remained significant in the multiple GLM, where submissions from 2022 to 2023 (RR: 1.85, 95 percent CI: 1.20–2.84, *p* = .005) demonstrated the strongest association with patient involvement.

The deliberation type was also a significant predictor. Discussion-type submissions demonstrated a higher patient involvement rate (35.4 percent) than reporting-type submissions (15.8 percent; *p* < .001). This association was confirmed in the simple GLM (RR: .45, 95 percent CI: .27–.73) and remained significant in the multiple GLM after adjusting for other factors (RR: .61, 95 percent CI: .39–.95; *p* = .029).

#### Disease area and application type

The disease area significantly influenced patient involvement rates. Submissions for rare diseases (39.3 percent) and oncology (29.9 percent) had the highest involvement, whereas those for autoimmune diseases (9.7 percent) and other catastrophic illnesses (14.3 percent) had the lowest (*p* = .040). The simple GLM confirmed lower participation for other catastrophic diseases (RR: .37, 95 percent CI: .16–.88) compared to oncology. This association remained significant in the multiple GLM (RR: .39, 95 percent CI: .17–.90; *p* = .027).

Application type also plays a critical role. Submissions for new drugs (53.7 percent) resulted in significantly higher patient involvement rates than those for new indications (6.8 percent; *p* < .001). This finding was supported by the simple GLM (RR: .14, 95 percent CI: .07–.27) and confirmed by the multiple GLM (RR: .14, 95 percent CI: .07–.27; *p* < .001).

#### Factors that lost significance in multiple GLM

Although the innovation category and the anticipated budget impact appeared significant in the chi-square analyses, these associations lost statistical significance in multiple GLMs. Initially, submissions classified as Category 1 (64.0 percent) and high-budget submissions (50 percent) exhibited the highest patient involvement rates; however, these associations were no longer significant after adjusting for multiple GLM.

#### Impact of the NHIA’s OPOP expansion on new indications (ad hoc analysis)

Following the NHIA’s October 2022 policy expansion to include new indications in OPOP, the patient involvement rate for new indications increased significantly from 1.9 percent to 24.1 percent (*p* = .00015). Logistic regression analysis confirmed that policy change strongly predicted patient involvement (OR, 2.79; *p* = .001), explaining 21.3 percent of the variance.

### Association between patient involvement and PBRS decisions

Among the 282 submissions, 261 (92.6 percent) were approved by PBRS. However, the presence of patient involvement (*n* = 74, 28.4 percent) was not significantly associated with the PBRS approval rate, which may reflect the inherent complexity of HTA decision-making or a limited influence of such involvement. The corresponding data are presented in Supplementary Appendix Table 5.

## Discussion

### Summary of key findings

This study reveals a substantial gap between policy intent and actual implementation of patient involvement in Taiwan’s pharmaceutical reimbursement decision-making process. Despite the NHIA’s established criteria for patient involvement through OPOP, only 28.4 percent of eligible submissions documented patient involvement over the study period. These findings confirm concerns raised by patient advocacy groups regarding incomplete inclusion of eligible catastrophic illness submissions in the OPOP, inconsistent incorporation of patient inputs into PBRS materials, and the limited observer role of patient representatives at PBRS meetings (31;32).

Encouragingly, our analysis identified a positive trend of increasing patient involvement, from 17 percent in 2016 to 44 percent in 2023. This growth trajectory reflects the NHIA’s gradual implementation of patient engagement policies alongside advocacy efforts from patient organizations and patient expert education programs (36). The Taiwan Alliance of Patients’ Organizations has played a particularly significant role in facilitating dialogue between patient groups and policymakers to advance patient involvement discussions in Taiwan.

### Interpretation through the mosaic model framework

The observed increase in patient involvement over time suggests a gradual enhancement of the mosaic of engagement in Taiwan’s pharmaceutical reimbursement system. However, when viewed through Tritter and McCallum’s mosaic model, our findings highlight an incomplete implementation of patient involvement mechanisms. The model emphasizes that effective patient involvement requires multiple, interconnected approaches tailored to different contexts (12). The significant variations across submission types in our study reveal substantial gaps in this mosaic, with patient involvement concentrated in high-profile submissions and later-stage decisions rather than distributed throughout the decision-making continuum.

This uneven distribution of patient involvement opportunities limits the potential for patients to meaningfully influence the reimbursement process. Although the NHIA has made progress in establishing consultation mechanisms through OPOP and allowing patient representatives to observe PBRS meetings, these represent isolated tiles within what should be a comprehensive mosaic of engagement. A more robust approach would integrate patient perspectives throughout the decision-making pathway, from initial submission review to final coverage determinations.

### Factors influencing patient involvement

Our analysis identified several key factors that significantly predict patient involvement in Taiwan’s reimbursement decisions. Deliberation type emerged as one of the strongest predictors, with discussion-type submissions demonstrating significantly higher patient involvement rate (35.4 percent) compared to reporting-type submissions (15.8 percent). This pattern indicates that the NHIA tends to incorporate patient opinions in cases requiring extensive policy deliberation rather than systematically engaging patients across all decision-making processes.

Disease area also significantly influenced patient involvement patterns, with oncology (29.9 percent) and rare disease submissions (39.3 percent) demonstrating significantly higher patient involvement rates, whereas autoimmune diseases (9.7 percent) and other catastrophic illnesses (14.3 percent) demonstrated markedly lower rates. This disparity likely reflects the strength of existing patient advocacy networks and public interest in certain disease categories, particularly cancer treatments.

The application type demonstrated perhaps the most striking difference, with new drug submissions (53.7 percent) resulting in significantly higher patient involvement rates than those for new indications (6.8 percent). This substantial gap highlights a critical structural barrier in Taiwan’s patient involvement framework that has only recently begun to be addressed through policy changes.

Interestingly, although budget impact and innovation categories initially appeared significant in our preliminary analyses, these associations lost statistical significance in the multiple GLMs. This suggests that their apparent influence was confounded by factors such as submission type or disease area. These findings align with international research, indicating that financial and innovation factors influence HTA processes but do not necessarily drive patient engagement unless submissions are explicitly prioritized for broader stakeholder consultation (37).

### Impact of policy changes on patient involvement

The significant increase in patient involvement for new indications following the NHIA’s October 2022 policy expansion provides compelling evidence that targeted policy interventions can effectively enhance patient involvement. Prior to this policy change, patient involvement rates for new indications were remarkably lower than those for new drugs, largely because new indications were previously excluded from the OPOP framework. The dramatic postpolicy increase from 1.9 percent to 24.1 percent reinforces the importance of addressing structural barriers to patient involvement through explicit policy reforms.

This finding has broader implications for future policy development, suggesting that similar targeted interventions could address other gaps identified in our analysis. However, given the relatively short post-policy timeframe in our study, further research will be necessary to assess whether this increased involvement is sustained over time and to identify additional mechanisms that might support long-term patient engagement across all submission types.

Despite these improvements in patient involvement rates, our study found no significant association between patient involvement and PBRS approval decisions. The remarkably high PBRS approval rate of 92.6 percent likely reflects the upstream impact of Expert Consultation Meetings (ECMs), where preliminary decisions on reimbursement feasibility are often made. Manufacturers typically revise or withdraw applications following unfavorable ECM recommendations, effectively filtering out submissions likely to be rejected before reaching the PBRS stage. This dynamic highlights a critical gap in Taiwan’s current framework: patient involvement is absent from these earlier, formative stages of decision-making where fundamental reimbursement criteria are shaped.

Although most patient opinions were either invited by the NHIA or presented in the PBRS meeting documents, only two submissions explicitly stated in the PBRS meeting records that patient input directly influenced the PBRS decisions. In April 2019, the PBRS meeting extended the reimbursement duration for Revolade® from 8 to 12 weeks for each patient based on patient feedback, despite an initial rejection due to concerns over clinical benefits and budget impact. Similarly, in February 2020, patient group advocacy led to continued evaluation of PD-1/PD-L1 immune checkpoint inhibitors based on real-world evidence. These cases highlight that although patient opinions can influence HTA decisions, such instances remain sporadic rather than systematic.

### International comparison of patient involvement implementation

When compared to international best practices, Taiwan’s approach to patient involvement reveals both progress and ongoing challenges. Countries like Canada, the UK, and Australia have implemented more comprehensive and earlier-stage patient engagement processes that can serve as valuable models for Taiwan’s evolving system.

Canada’s CADTH-pCODR allows patient input upon manufacturer submission notifications, ensuring that feedback is collected and validated with patient groups before deliberation begins. Moreover, patient representatives hold voting rights on the pERC (pan-Canadian Oncology Drug Review Committee), enabling direct influence on final reimbursement decisions (38). This approach stands in contrast to Taiwan’s current observer-only role for patient representatives.

Similarly, the UK’s NICE incorporates patient perspectives throughout the HTA process, from initial scoping to final decisions, ensuring continuous engagement rather than limiting inputs to late-stage consultations (2). Australia’s PBAC engages patient representatives not only through online platforms but also via consumer hearings and stakeholder meetings, allowing real-time discussions with decision-makers (39).

These international comparisons highlight opportunities for Taiwan to strengthen its patient involvement framework by expanding engagement opportunities beyond the PBRS meeting stage. The experiences of these countries demonstrate that meaningful patient involvement requires multiple interconnected mechanisms distributed throughout the decision-making process.

### Policy implications

Based on our findings and international comparisons, several policy recommendations emerge for enhancing patient involvement in Taiwan’s pharmaceutical reimbursement decisions. Aligning with the mosaic model (12), Taiwan’s NHIA would benefit from expanding patient involvement beyond consultation to inform PBRS meetings to create a more comprehensive engagement framework.

Establishing flexible and continuous mechanisms – such as proactive patient outreach, diverse advisory groups, and inclusive deliberation methods – could systematically integrate the full spectrum of patient voices and experiences into decision-making processes. Additionally, automating OPOP listing processes would ensure consistent application of involvement criteria across all eligible submissions, addressing the current implementation gaps identified in our study.

A particularly important reform would be incorporating patient representatives into earlier decision stages, such as ECMs, where fundamental reimbursement criteria are shaped. Existing research indicates that engaging patients earlier in the decision-making process enhances their influence, whereas participation closer to final decisions often has a more limited impact (13;40). Although our study found that patient input may not directly alter final PBRS decisions, earlier involvement could provide valuable context, reassurance, and insights that ultimately enrich decision-making quality (9;41).

The demonstrated interest of Taiwan’s patient advocacy groups in contributing to a fair healthcare environment presents a valuable opportunity for policy development. Notably, three patient-involved submissions were excluded from this study because they fell outside the predefined OPOP scope of NHIA. These cases occurred in June 2017, April 2010, and October 2023, involving administrative adjustments, expanding coverage for immune thrombocytopenia, and health technology reassessment for orphan drugs, respectively. These cases demonstrate patients’ desire for broader involvement beyond initial coverage decisions for catastrophic illnesses and underscore the necessity of further expanding patient involvement opportunities across the full spectrum of reimbursement decision-making.

### Strengths and limitations

This study represents the first systematic examination of the gap between policy and practice in Taiwan’s patient involvement implementation, focusing specifically on the NHIA’s role in facilitating patient involvement rather than the depth or quality of patient contributions. This focus aligns with our objective of assessing institutional implementation rather than patient-driven engagement. Although prior research has explored patient involvement in European and American healthcare systems, empirical studies on patient involvement within East Asian healthcare systems – particularly under single-payer models, such as the NHI in Taiwan – remain limited. By addressing this gap, this study contributes to global discussions on best practices for patient involvement in reimbursement decision-making.

A key strength of this study lies in its deliberative validation process, where field experts reviewed and cross-validated the submission data to ensure accuracy and reliability in capturing real-world reimbursement practices. The comprehensive data set spanning from 2016 to 2023 allows for meaningful trend analysis and policy impact assessment that would not be possible with a more limited timeframe.

However, this study is limited by the unavailability of raw data from the OPOP, which necessitated reliance on secondary sources to define patient involvement. The study also focuses primarily on quantitative measures of involvement rather than qualitative assessment of patient input quality or impact. Future research could explore the qualitative aspects of patient engagement and investigate whether early-stage patient involvement such as during ECMs, influences decision outcomes differently than later-stage consultation. Additionally, longer-term follow-up on the impact of the 2022 policy expansion would provide valuable insights into the sustainability of policy-driven improvements in patient involvement rates.

## Conclusions

This study identified significant gaps between Taiwan’s patient involvement policy and its real-world execution, highlighting low overall engagement and disparities across deliberation types and disease areas. The success of NHIA 2022 policy expansion demonstrates that targeted interventions can effectively improve patient involvement rates, reinforcing the need for broader structural reforms to make patient involvement more inclusive and impactful.

Viewing Taiwan’s patient involvement framework through the mosaic model lens highlights both the progress made and the opportunity to develop a more comprehensive, interconnected system of engagement that acknowledges different patient experiences, incorporates diverse expertise, and fosters dynamic stakeholder relationships throughout the reimbursement process.

Policymakers should prioritize establishing structured mechanisms for incorporating patient input into reimbursement decision-making, ensuring broader participation beyond high-profile submissions. Expanding patient engagement strategies beyond PBRS meetings, such as formalizing patient advisory roles or involving patient representatives in ECMs, could further strengthen the integration of patient perspectives in shaping coverage decisions. Taiwan’s reimbursement framework must evolve to ensure that all stakeholders, particularly underrepresented patient groups, have an equitable voice in decision-making.

The evolving patient involvement landscape in Taiwan presents opportunities for international collaboration and policy learning. Strengthening patient engagement not only enhances transparency and decision legitimacy but also addresses the paucity of empirical research on patient involvement in East Asian healthcare systems. As countries worldwide refine their patient-centered health policies, Taiwan’s experiences could serve as valuable case studies for improving patient representation in reimbursement decision-making.

## Supporting information

Tsai et al. supplementary material Tsai et al. supplementary material

## Data Availability

The data sets analyzed during the current study are available upon reasonable request from the corresponding author.

## Supplementary material

The supplementary material for this article can be found at http://doi.org/10.1017/S026646232510038X.
