# Supplementary material for: Evaluation of implementation of patient involvement in Taiwan’s pharmaceutical reimbursement decision-making process
Source: Int J Technol Assess Health Care. 2025 Jul 29;41(1):e55. doi: 10.1017/S026646232510038X (PMC12350087; doi:10.1017/S026646232510038X)
Supplement: Tsai et al. supplementary material [file S026646232510038Xsup001.pdf]

Appendix 1 The criteria of reporting type of submission

A **reporting-type submission** refers to a reimbursement application that meets **specific NHIA criteria**, allowing **expedited approval without extensive deliberation**. The criteria for reporting the type of submission are as follows:

- New drugs that share the same first five digits of the Anatomical Therapeutic Chemical (ATC) classification as drugs already listed exhibit similar therapeutic efficacy and indications and have a per-treatment course cost equal to or less than that of the reference products included in the NHI reimbursement list.
- New combination drugs, where each individual component is already covered by NHI reimbursements and the combined cost per treatment course does not exceed the total cost of the individual components when administered separately.

The NHIA considers that these criteria for **reporting-type submissions as not requiring additional budget impact considerations or major clinical benefit assessments**, thereby streamlining the reimbursement process.

Source: NHIA, *Principles for listing submissions under the Pharmaceutical Benefit and Reimbursement Scheme (PBRS) reporting type.* 2017: Report presented at the 24th PBRS Joint Committee Meeting (Drug Section), item 1 of the meeting report.

Appendix 2 Earmarked budget in Taiwan's NHI system

Pharmaceutical expenditure in Taiwan is funded through two sources: the general budget and earmarked funds. The general budget covers a broad range of medical services. Because of the global budget cap in Taiwan's National Health Insurance (NHI) system, an increase in pharmaceutical spending could potentially reduce funding for other healthcare services. In contrast, earmarked funds are specifically allocated to certain services or medications. When a drug is funded through an earmarked budget, its expenditure is restricted to a designated fund and does not affect the budgets for other medical services.

Comparison of General Budget vs. Earmarked Budget in Taiwan's NHI System

Category	General Budget	Earmarked Budget
**Funding Source**	Part of the overall NHI budget, shared across all medical services.	Part of the overall NHI budget, dedicated funds allocated for specific medications or services.
**Scope**	Covers a broad range of medical services, including pharmaceuticals.	Restricted to particular conditions, such as rare diseases or specific treatments.
**Impact on Other Services**	Subject to the global budget cap; increased pharmaceutical spending may reduce funding for other services.	Does not impact funding for other medical services.
**Budget Flexibility**	More flexible but constrained by the overall NHI budget cap.	Less flexible; funds are pre-allocated and cannot be diverted to other expenses.
**Purpose**	Used to reimburse medications alongside other healthcare expenditures.	Ensures funding availability for designated medications without competing with general healthcare needs.

Appendix 3 Approach and Explanation for Addressing Issues Encountered During Data Validation.

**Issue**	**Consensus among experts_**	**Note**
The same case may undergo multiple PBRS meetings, leading to ambiguity in annual categorization.	Cases will be categorized based on the PBRS meeting resolution of the respective year.	Repeated counting of the same case was considered to occur when a case is included in the case count despite not reaching a resolution.
How is the case considered "approved" by PBRS?	Cases are considered approved if they have received approval during the PBRS meeting, even if the pricing and reimbursement scope differ from the applicant's request.	PBRS often agrees on pricing and reimbursement scope different from the applicant's request.
What were the criteria for inclusion and exclusion of cases?	Detailed inclusion and exclusion criteria, as determined by consensus, were outlined in manuscripts.	A consensus was reached after reviewing all cases.
How should indications be classified?	Indications were not further subdivided, e.g., breast cancer will not be categorized as first-line or second-line treatment, and distinctions between metastatic or adjuvant therapy are not made.	Further subclassification of indications would be overly complex.
What is the approach for situations with multiple drugs discussing the same indication simultaneously?	Case number was counted on a per-drug basis, even if there are multiple drugs for the same indication within one discussion. Each drug was treated as an independent case. regardless of the number of indications per individual drug.	Experts agreed that using drugs as the basis for case determination is the most reasonable approach.
When cases span different disease categories, how should they be categorized?	Cases were categorized under the disease category with the most indications discussed for that drug, following a consensus decision. For example, a certain drug with 7 indications under solid tumor and one indication under hematology, was categorized into solid tumor case.	Consensus decision
When cases involve drugs falling under exclusion criteria, yet the meeting documents presented patient group correspondence, how should they be treated?	We decided to exclude them from the analysis, following a consensus decision.	Consensus decision

Appendix 4. Factors contributing to patient involvement in simple and multiple GLMs

	Simple GLM		Multiple GLM	
**Charteristics**	**RR (95%CI)**	**p-value**	**ARR (95%CI)**	**p-value**
Year of PBRS Conclusion				
2016-2018	1 (reference)		1 (reference)	
2019-2021	1.29 (0.77-2.17)	0.3332	1.48 (0.95-2.30)	0.0837
2022-2023	1.94 (1.17-3.21)	0.0100	1.85 (1.20-2.84)	0.005
Deliberation Type				
Discussion type of submission	1 (reference)		1 (reference)	
Reporting type of submission	0.45 (0.27-0.73)	0.0013	0.61 (0.39-0.95)	0.029
Disease Area				
Oncology	1 (reference)		1 (reference)	
Hemophilia and rare disease	1.17 (0.80-1.71)	0.4235	0.84 (0.61-1.14)	0.2599
Other catastrophic disease	0.37 (0.16-0.88)	0.024	0.39 (0.17-0.90)	0.0266
Application Type				
New drugs	1 (reference)		1 (reference)	
New indications	0.14 (0.07-0.27)	<.0001	0.14 (0.07-0.27)	<.0001
Innovation Category				
1	1 (reference)		-	-
2A	0.85 (0.58-1.23)	0.3883	-	-
2B	0.55 (0.36-0.86)	0.0084	-	-
not available	0.11 (0.05-0.21)	<.0001	-	-
Anticipated Budget Impact				
≦100 million or no data	1		-	-
>100 million	2.03 (1.42-2.91)	<.0001	-	-

GLM=generalized linear model; RR= Relative Risk; ARR=Adjusted Relative Risk; CI: confidence Interval.

The results from multiple GLMs are presented only for factors that achieved statistical significance ($p < 0.05$).

Appendix 5. Association Between Patient Involvement and PBRS Decisions

PBRS decision	Submissions with patient involvement	Submissions without patient involvement	Column total	*p-value*
Positive decision	74 (28.4%)	187 (71.6%)	261	0.98
Negative decision	6 (28.6%)	15 (71.4%)	21	
Row total	80 (28.4%)	202 (71.6%)	282	
$\chi 2$= 0.00046				

Appendix Figure 1. Characteristics of the investigated submissions. Figure 1A presents the deliberation types of PBRS Meeting submissions for each year. Figure 1B presents the application types of PBRS Meeting submissions for each year. Figure 1C shows the disease areas of the PBRS that meet the submissions for each year. Figure 1D presents the anticipated budget impact in the fifth reimbursement year of the PBRS Meeting submissions. Figure 1E shows the innovation categories of new drug submissions for each year. Figure 1F shows the decisions of the PBRS meetings for each year.

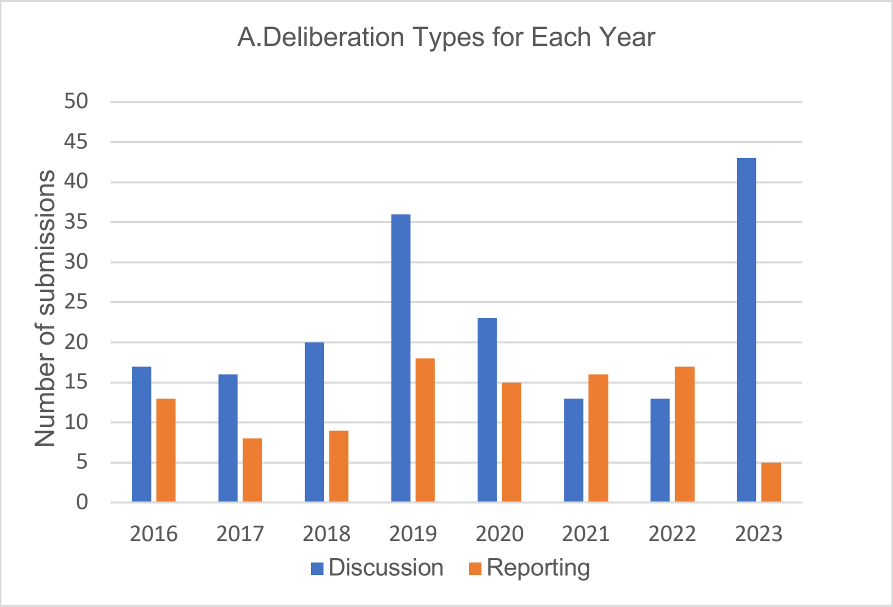

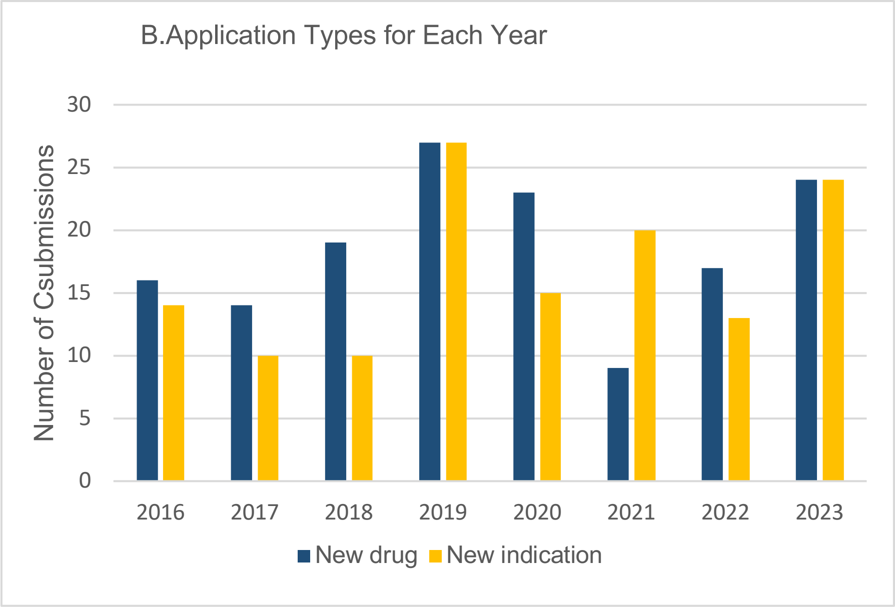

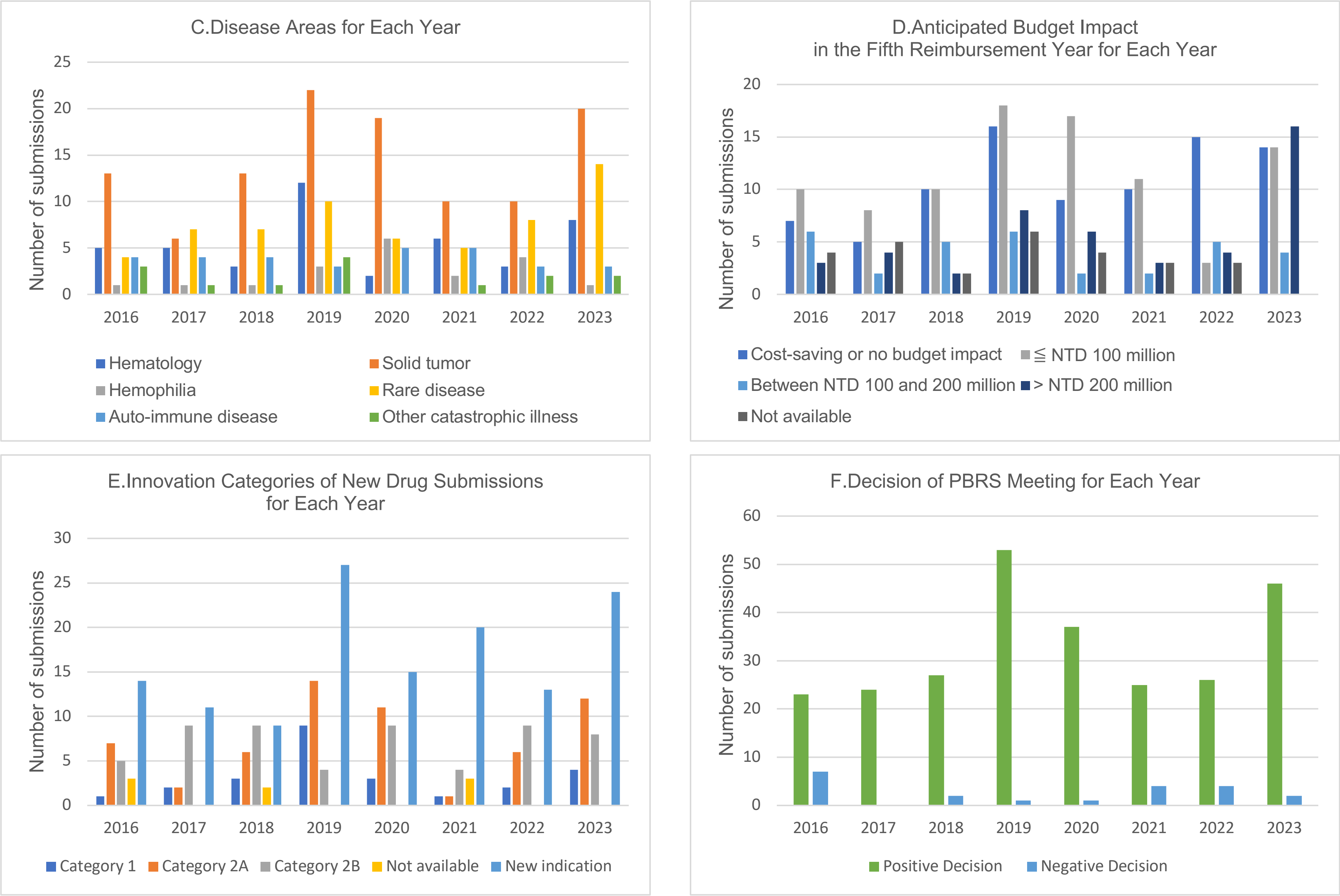
C.Disease Areas for Each Year
Number of submissions
0
5
10
15
20
25
2016
2017
2018
2019
2020
2021
2022
2023
Hematology
Solid tumor
Hemophilia
Rare disease
Auto-immune disease
Other catastrophic illness
D.Anticipated Budget Impact
in the Fifth Reimbursement Year for Each Year
Number of submissions
0
5
10
15
20
2016
2017
2018
2019
2020
2021
2022
2023
Cost-saving or no budget impact
≦ NTD 100 million
Between NTD 100 and 200 million
> NTD 200 million
Not available
E.Innovation Categories of New Drug Submissions
for Each Year
Number of submissions
0
5
10
15
20
25
30
2016
2017
2018
2019
2020
2021
2022
2023
Category 1
Category 2A
Category 2B
Not available
New indication
F.Decision of PBRS Meeting for Each Year
Number of submissions
0
10
20
30
40
50
60
2016
2017
2018
2019
2020
2021
2022
2023
Positive Decision
Negative Decision
